# Tularemia — United States, 2011–2022

**DOI:** 10.15585/mmwr.mm735152a1

**Published:** 2025-01-02

**Authors:** Shannan N. Rich, Alison F. Hinckley, Austin Earley, Jeannine M. Petersen, Paul S. Mead, Kiersten J. Kugeler

**Affiliations:** ^1^Division of Vector-Borne Diseases, National Center for Emerging and Zoonotic Infectious Diseases, CDC; ^2^Epidemic Intelligence Service, CDC.

**Summary**

**What is already known about this topic?**

Tularemia, a rare bacterial zoonotic disease*,* can lead to death but is treatable with antibiotics. It is caused by the tier-1 select agent *Francisella tularensis*, which can be transmitted to humans through multiple routes.

**What is added by this report?**

During 2011–2022, 47 states reported 2,462 tularemia cases (0.064 per 100,000 population), representing a 56% increase in incidence compared with 2001–2010. Incidence was highest among children aged 5–9 years, older men, and American Indian or Alaska Native persons, among whom incidence was approximately five times that among White persons.

**What are the implications for public health practice?**

Increased tularemia incidence might reflect changes in frequency of human infection or improved case ascertainment. Reducing incidence will require tailored prevention strategies and health care provider education.

## Abstract

Tularemia is a rare nationally notifiable zoonosis, caused by the tier-1 select agent *Francisella tularensis,* that has been reported from all U.S. states except Hawaii. Clinical manifestations typically include fever and localized symptoms that vary by route of infection. The case fatality rate of tularemia is typically <2% but can be higher depending on clinical manifestation and infecting strain. Tularemia is treatable with antibiotics. During 2011–2022, a total of 47 states reported 2,462 tularemia cases, but four central states (Arkansas, Kansas, Missouri, and Oklahoma) accounted for 50% of all reported cases. Incidence was highest among children aged 5–9 years (0.083 per 100,000 population) and adult males aged 65–84 years (range = 0.133–0.161). Incidence among American Indian or Alaska Native persons (0.260) was approximately five times that among White persons (0.057). The average annual incidence of tularemia in the United States during 2011–2022 (0.064) was 56% higher than that reported during 2001–2010 (0.041), largely resulting from increased reporting of probable cases. These findings might reflect an actual increase in human infection or improved case detection amid changes in commercially available laboratory tests during this period. Reducing tularemia incidence will require tailored prevention education; mitigating morbidity and mortality will require health care provider education, particularly among providers serving tribal populations, regarding early and accurate diagnosis and treatment.

## Introduction

Tularemia is a rare bacterial zoonotic disease caused by *Francisella tularensis,* an organism that has been designated a tier-1 select agent based on its potential for misuse as a bioweapon (*1*). No vaccine to prevent tularemia is currently available. Human infection can occur naturally through an arthropod bite (e.g., deer flies and ticks), improper handling of infected animals, inhaling contaminated aerosols, or drinking contaminated water (*2*). Clinical manifestations typically include fever and localized symptoms that vary by route of infection, such as skin ulcers, regional lymphadenopathy, and pneumonia. Tularemia is treatable with antibiotics. The case fatality rate of tularemia is typically <2% (*3*) but can be as high as 24%, depending on the infecting genotype and clinical manifestation (*4*). Tularemia is a nationally notifiable disease in the United States; cases are reported by state health departments to CDC through the National Notifiable Diseases Surveillance System. This report summarizes U.S. tularemia surveillance data reported during 2011–2022.

## Methods

### Case Definition

For surveillance purposes, a confirmed case of tularemia is defined as clinically compatible illness with isolation of *F. tularensis* in culture, or a fourfold change in antibody titer between acute and convalescent serum samples.* A probable case is defined as clinically compatible illness with a single elevated serologic antibody titer or detection of *F. tularensis* in a clinical specimen by fluorescent assay.^†^ In 2017, laboratory criteria for a probable case were expanded to also include detection of *F. tularensis* by polymerase chain reaction.

### Calculation of Incidence

Annual incidence of tularemia was calculated as the number of cases per 100,000 population using United States Census Bureau population estimates for 2017 (*5*). Average annual incidence during 2011–2022 was calculated overall, by demographic characteristics, case classification (i.e., confirmed or probable), and state. This activity was reviewed by CDC, deemed not research, and was conducted consistent with applicable federal law and CDC policy.^§^

## Results

### Reported Tularemia Cases

**FIGURE 1:**
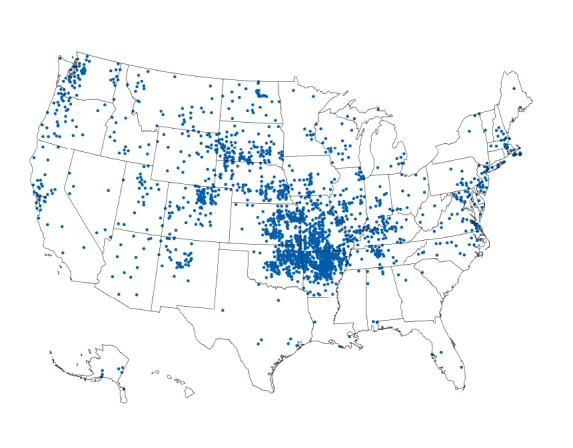
Reported tularemia cases, by county of residence* — United States, 2011–2022 * Cases are indicated randomly within county of residence.

During 2011–2022, a total of 2,462 tularemia cases were reported in the United States, including 984 (40%) confirmed and 1,475 (60%) probable cases. A mean of 205 cases was reported per year, ranging from 149 in 2012 to 314 in 2015. Overall average annual incidence was 0.064 cases per 100,000 population. Cases were reported among residents of 743 counties in 47 states (Figure 1). Four central states accounted for 50% of all reported cases: Arkansas (18%), Kansas (11%), Missouri (11%), and Oklahoma (10%) (Supplementary Table, https://stacks.cdc.gov/view/cdc/174815). A majority of patients (78%) were reported to have symptom onset during May–September.

### Demographic Characteristics of Cases

The median patient age was 48 years (range = 1–95 years); 63% of patients were male. White persons accounted for most cases (84%), followed by American Indian or Alaska Native (AI/AN) (9%), Black or African American (2%), and Asian or Pacific Islander (1%) persons; 5% of patients identified as Hispanic or Latino.

### Tularemia Incidence

**FIGURE 2:**
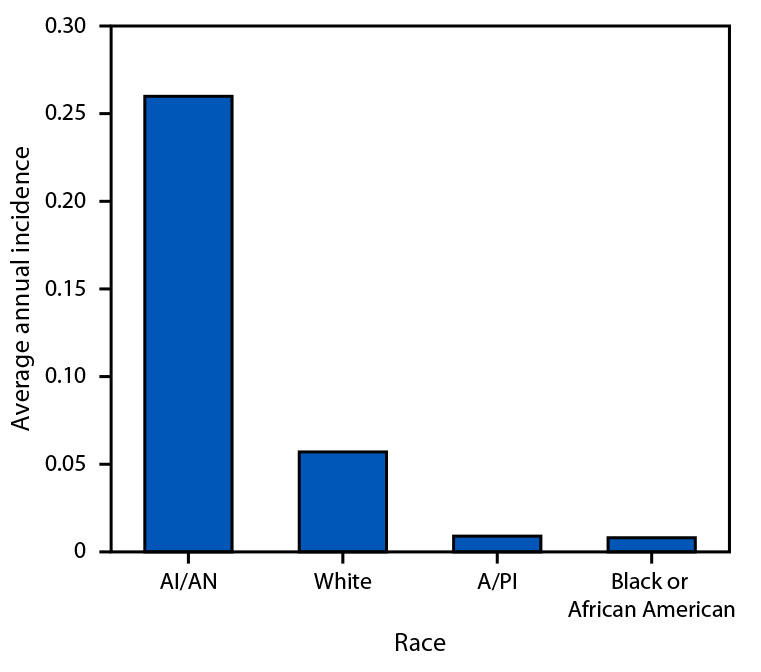
Average annual tularemia incidence,* by race — United States, 2011–2022 **Abbreviations:** AI/AN = American Indian or Alaska Native; A/PI = Asian or Pacific Islander. * Cases per 100,000 population.

Among racial groups, tularemia incidence was highest among AI/AN persons (0.260 per 100,000 population) (Figure 2). By age group and sex, incidence was highest among children aged 5–9 years (0.083) and adult males, particularly those aged 65–84 years (range = 0.133–0.161) (Supplementary Figure, https://stacks.cdc.gov/view/cdc/174814).

**FIGURE 3:**
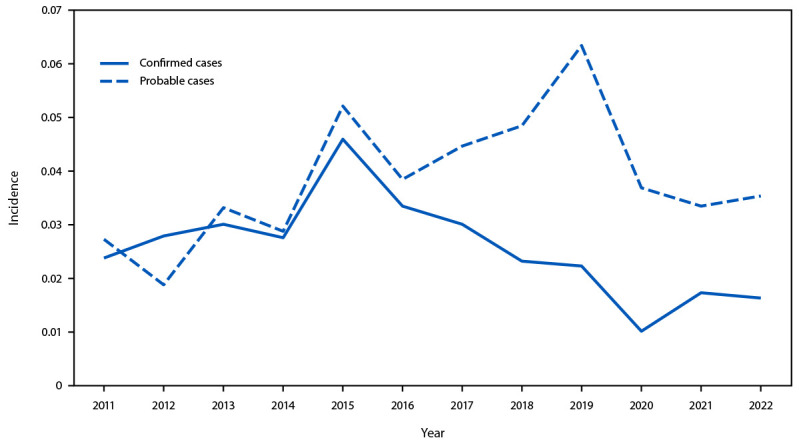
Tularemia incidence,* by case status^†^ and year — United States, 2011–2022 * Cases per 100,000 population. ^†^ A confirmed case of tularemia is defined as clinically compatible illness with isolation of *Francisella tularensis* in culture, or a fourfold change in antibody titer between acute and convalescent serum samples. A probable case is defined as clinically compatible illness with a single elevated serologic antibody titer or detection of *F. tularensis* in a clinical specimen by fluorescent assay. In 2017, laboratory criteria for a probable case were expanded to also include detection of *F. tularensis* by polymerase chain reaction.

During 2011–2022, the average annual incidence of probable cases of tularemia (0.038 per 100,000 population) exceeded that of confirmed cases of tularemia (0.026). Incidence of probable cases has exceeded that of confirmed cases consistently since 2015, and temporal trends in confirmed and probable incidence diverged from one another beginning in 2017 (Figure 3). The highest incidence of probable tularemia was among children aged 5–9 years (0.052 per 100,000 population); AI/AN persons (0.185); and persons who lived in the central U.S. states of Arkansas (1.073), South Dakota (0.642), Kansas (0.432), and Oklahoma (0.367) (Supplementary Table, https://stacks.cdc.gov/view/cdc/174815).

## Discussion

The demographic characteristics and geographic distribution of tularemia patients in the United States during 2011–2022 were comparable to those described during 2001–2010, with the highest incidence observed among children aged 5–9 years, older men, AI/AN persons, and persons living in the central United States (*6*). AI/AN persons remain the demographic group most affected by tularemia, with incidence in this group approximately five times that among White persons. Many factors might contribute to the higher risk for tularemia in this population, including the concentration of Native American reservations in central states and sociocultural or occupational activities that might increase contact with infected wildlife or arthropods.

The average annual tularemia incidence during 2011–2022 (0.064 cases per 100,000 population) was 56% higher than that reported during 2001–2010 (0.041) (*6*). Notable differences in case classification were also apparent compared with the previous decade: during 2011–2022, 60% of reported cases were classified as probable, a 71% increase compared with the 35% of cases classified as probable during 2001–2010. The annual incidence of probable cases of tularemia was increasing before the 2017 case definition change that included detection of *F. tularensis* by polymerase chain reaction in the probable case definition, indicating that updated surveillance criteria do not fully explain the trend. Increased reporting of probable cases might be associated with an actual increase in human infection, improved tularemia detection, or both. In addition, in recent years, some commercial laboratories have shifted from agglutination assays to enzyme-linked immunosorbent assays that are more sensitive and less specific (*7*). These newer assays do not generate titers that can be readily compared between acute and convalescent specimens to infer acute infection. Consequently, cases that previously would have been classified as confirmed based on a fourfold change in titer, would instead be classified as probable cases when tested by enzyme-linked immunosorbent assays.

### Limitations

The findings in this report are subject to at least three limitations. First, CDC does not receive clinical and laboratory details for most reported cases, limiting the ability to ascertain the drivers of the observed patterns. This information is provided to CDC by state health departments on a voluntary basis and for a minority of reported cases (*8*). Second, surveillance practices differ by state and have changed over time; thus, these data might not represent all U.S. tularemia infections. Finally, the COVID-19 pandemic might have affected the ability of health departments to classify possible tularemia cases.

### Implications for Public Health Practice

Risk for tularemia persists throughout the United States, particularly in some central states. Although the demographic groups most at risk for tularemia remain consistent with those identified in previous decades, AI/AN persons continue to face substantially higher disease risk than do other groups. 

The findings in this report highlight the need for tailored prevention education given the myriad of potential exposures to *F. tularensis* in the environment, including via inhalation, ingestion, contact with animals, or arthropod bites. In addition, increasing tularemia incidence underscores ongoing needs for clinical education regarding diverse clinical manifestations of tularemia, options for laboratory testing *(**9**)*, and importance of early and appropriate treatment with aminoglycoside, fluoroquinolone, or tetracycline classes of antimicrobials *(**3**)*, particularly for health care providers serving tribal populations. Although the signs and symptoms of tularemia are broad and often nonspecific, health care providers should consider tularemia in patients with clinically compatible illness (e.g., fever accompanied by lymphadenopathy) after possible exposure to *F. tularensis*, and laboratories should be alerted to suspicion of tularemia when possible to enable specific diagnostic considerations and ensure appropriate safety precautions *(**9**)*.

## Footnotes

*
https://ndc.services.cdc.gov/case-definitions/tularemia-2017/

†
https://ndc.services.cdc.gov/case-definitions/tularemia-1999/

§ 45 C.F.R. part 46.102(l)(2), 21 C.F.R. part 56; 42 U.S.C. Sect. 241(d); 5 U.S.C. Sect. 552a; 44 U.S.C. Sect.3501 et seq.
